# The Barrier Disruption and Pyroptosis of Intestinal Epithelial Cells Caused by Perfringolysin O (PFO) from *Clostridium perfringens*

**DOI:** 10.3390/cells13131140

**Published:** 2024-07-03

**Authors:** Zhankui Liu, Shuang Mou, Liang Li, Qichao Chen, Ruicheng Yang, Shibang Guo, Yancheng Jin, Lixinjie Liu, Tianzhi Li, Huanchun Chen, Xiangru Wang

**Affiliations:** 1 National Key Laboratory of Agricultural Microbiology, College of Veterinary Medicine, Huazhong Agricultural University, Wuhan 430070, China; zkliu1996@webmail.hzau.edu.cn (Z.L.); shuangmou@webmail.hzau.edu.cn (S.M.); liangli@webmail.hzau.edu.cn (L.L.); chenqc@webmail.hzau.edu.cn (Q.C.); yangruicheng@mail.hzau.edu.cn (R.Y.); shibangguo@webmail.hzau.edu.cn (S.G.); jinyancheng@webmail.hzau.edu.cn (Y.J.); liulxj@webmail.hzau.edu.cn (L.L.); litianzhi@webmail.hzau.edu.cn (T.L.); chenhch@webmail.hzau.edu.cn (H.C.); 2 Key Laboratory of Preventive Veterinary Medicine in Hubei Province, The Cooperative Innovation Center for Sustainable Pig Production, Wuhan 430070, China; 3 Engineering Research Center of Animal Biopharmaceuticals, The Ministry of Education of the People’s Republic of China (MOE), Wuhan 430070, China; 4 Frontiers Science Center for Animal Breeding and Sustainable Production, Wuhan 430070, China

**Keywords:** *C. perfringens* type C, *Clostridium perfringens* Theta toxin, pyroptosis, IPEC-J2, intestinal barrier

## Abstract

*Clostridium perfringens* (*C. perfringens*), a Gram-positive bacterium, produces a variety of toxins and extracellular enzymes that can lead to disease in both humans and animals. Common symptoms include abdominal swelling, diarrhea, and intestinal inflammation. Severe cases can result in complications like intestinal hemorrhage, edema, and even death. The primary toxins contributing to morbidity in *C. perfringens*-infected intestines are CPA, CPB, CPB2, CPE, and PFO. Amongst these, CPB, CPB2, and CPE are implicated in apoptosis development, while CPA is associated with cell death, increased intracellular ROS levels, and the release of the inflammatory factor IL-18. However, the exact mechanism by which PFO toxins exert their effects in the infected gut is still unidentified. This study demonstrates that a *C. perfringens* PFO toxin infection disrupts the intestinal epithelial barrier function through in vitro and in vivo models. This study emphasizes the notable influence of PFO toxins on intestinal barrier integrity in the context of *C. perfringens* infections. It reveals that PFO toxins increase ROS production by causing mitochondrial damage, triggering pyroptosis in IPEC-J2 cells, and consequently resulting in compromised intestinal barrier function. These results offer a scientific foundation for developing preventive and therapeutic approaches against *C. perfringens* infections.

## 1. Introduction

*C. perfringens* is widely distributed in the environment [1], being present in various sources such as soil, dust, feces, feed, raw meat, poultry litter, sewage, and human and animal intestines. It is acknowledged as a notable foodborne pathogen [2,3,4,5,6,7]. *C. perfringens* has the ability to infect both humans and animals, presenting a significant risk to their well-being [8]. Common symptoms of a *C. perfringens* infection typically involve abdominal swelling, diarrhea, and intestinal inflammation. If not addressed promptly, severe intestinal inflammation can lead to complications such as intestinal hemorrhage, edema, and potentially fatal consequences [9]. The development of intestinal diseases due to *C. perfringens* is primarily linked to the release of exotoxins and lytic enzymes, such as CPA, CPB, CPE, CPB2, perfringolysin O(PFO), and zmpa, etc. [10]. 

The intestine is commonly referred to as the body’s ‘second brain’ and is home to the largest component of the body’s digestive and immune system [11,12]. Comprised of intestinal epithelial cells, the intestinal barrier acts as the body’s primary digestive component and plays a critical role in the digestion and absorption of nutrients [13]. Serving as the main defense against the entry of bacteria and toxins from the gut into the bloodstream, maintaining the integrity of the intestinal epithelial barrier is crucial for gut homeostasis and overall host health [14,15]. Tight junctions (TJs) are equally important for preserving barrier integrity and are essential for maintaining intestinal homeostasis [16]. Impairment of the intestinal epithelial barrier, caused by factors such as bacterial toxins, viruses, environmental pollutants, and pesticide residues, is a significant contributor to gastrointestinal diseases [17,18,19,20]. Damage to the intestinal barrier leads to increased permeability, bacterial translocation, exacerbation of intestinal integrity injury, and subsequent disruption of normal physiological functions [21]. 

Intestinal necrosis and dysfunction resulting from *C. perfringens* infections are primarily due to secreted toxins CPA, CPB, CPB2, and CPE. The binding of the CPA toxin to GM1 enables the interaction with the pro-myosin receptor kinase A (TrKA), triggering the activation of the MEK/ERK pathway and ultimately causing cell death [22]. CPB toxin also induces necrosis and apoptosis but is highly susceptible to trypsin and other intestinal proteases, necessitating trypsin inhibitors for in vivo activity maintenance [23]. CPE toxin binds to claudins, forming a complex pore in the cell membrane that allows cations like Ca^2+^ to enter the cell, resulting in apoptosis and cell necrosis [24,25]. The PFO toxin gene is located on the *C. perfringens* chromosome and is thought to be essential for gas gangrene development in humans [26,27]. However, the potential of PFO to damage the intestinal epithelial barrier remains unclear, warranting further investigation to elucidate this mechanism.

Recent studies have highlighted the role of pyroptosis in intestinal barrier damage. *C. perfringens* toxins, particularly the PFO toxin, have been found to induce pyroptosis by activating the NLRP3 inflammasome [28]. Further investigation is required to ascertain whether the PFO toxin specifically initiates pyroptosis in intestinal epithelial cells.

This study examined the impact of the *C. perfringens* PFO toxin on intestinal epithelial barrier function using both the transwell model and a mouse model. Our findings highlight the significant role of the PFO toxin in disrupting the intestinal barrier during *C. perfringens* infection and provide insight into the mechanism through which the toxin induces intestinal barrier dysfunction by triggering pyroptosis. Our study illustrates the impact of *C. perfringens* infection on intestinal barrier function, offering scientific data and a theoretical framework to enhance understanding of the pathogenic mechanism. This research establishes a solid foundation for the future prevention and control of *C. perfringens*.

## 2. Materials and Methods

### 2.1. Cell Culture and Treatment

The porcine intestinal epithelial cells (IPEC-J2 cells, DSMZ ACC-701) were cultured in Dulbecco’s Modified Eagle’s Medium (DMEM), supplemented with antibiotics (100 units/mL penicillin and 100 mg/L streptomycin) and 10% fetal bovine serum. The culture was incubated at 37 °C with a 5% CO_2_ atmosphere. For the experiments, IPEC-J2 cells (1 × 10^5^) were seeded into six-well tissue-culture plates containing complete media supplemented with 10% FBS. Once the cells reached 80–90% confluence in the culture plates, different wells were treated with varying concentrations of PFO (0.1, 0.2, 0.5, and 1.0 mg/L) for a duration of 30 min. It is crucial to ensure full cell confluence to accurately observe tight junctions between cells in the culture plate.

### 2.2. Antibodies, Reagents, and Plasmids

The ZO-1 (rabbit) antibody (21773-1-AP), Claudin-1 (rabbit) antibody (13050-1-AP), β-actin (mouse) antibody (66009-1-Ig), anti-6*His-Tag (rabbit) antibody (66005-1-Ig), and IL-1β (rabbit) antibody (16806-1-AP) were obtained from Proteintech Group (Chicago, IL, USA). The secondary antibody used for immunofluorescence was Cy3-conjugated Affinipure Goat Anti-Rabbit IgG(H+L) (SA00009-2) also sourced from Proteintech Group. The GSDMDC1 (mouse) antibody (sc-81868) was procured from Santa Cruz Biotechnology (Dallas, CA, USA). For immunoblotting analysis, horseradish peroxidase (HRP)-conjugated anti-rabbit IgG (BF03008) and HRP-conjugated anti-mouse IgG (BF03001) were acquired from Biodragon (Beijing, China). The inhibitors Z-VAD-FMK (HY-16658B) and N-Acetyl-L-cysteine (HY-B0215) were purchased from MedChemExpress (Monmouth Junction, NJ, USA). The pCold™ I DNA (3361) was obtained from Takara Biomedical Technology (Dalian, China).

### 2.3. Expression and Purification of PFO

We employed the pCold-I vector to facilitate robust expression of cloned genes upon induction by cold shock. Initially, the *PFO* gene from *C. perfringens* (C59-2) was amplified via PCR and inserted into pCold-I, resulting in the creation of pCold-I-*PFO.* The constructed plasmid was then introduced into *Escherichia coli* strain BL21 (DE3) and cultured at 37 °C with agitation. Once reaching an OD_600_ of 0.4–0.5, the culture was rapidly cooled to 15 °C in ice water for a duration of 30 min, followed by the addition of isopropyl β-D-thiogalactoside to achieve a final concentration of 1.0 mM. Subsequently, the culture was maintained at 15 °C for a period of 24 h. Analysis of bacterial protein samples for PFO expression was conducted using 12% SDS-PAGE gel electrophoresis. The recombinant protein was purified utilizing a Ni-NTA Superflow cartridge (Qiagen) and eluted with imidazole as an elution buffer component. Fractionation analysis through SDS-PAGE confirmed the presence of recombinant PFO at its expected size (∼55 kDa). The most enriched fractions were pooled together, concentrated, desalted, and stored in aliquots at −80 °C in a solution containing 20 mM NaCl, 25 mM HEPES (pH 7.2), and 5% glycerol until further use. Protein concentration determination was determined using the BCA protein concentration determination kit (Biosharp, Beijing, China).

### 2.4. Animal Experiments and Sample Collection

Healthy 3- to 4-week-old female BALB/c mice (11 ± 1 g in body weight) were obtained from the Laboratory Animal Center of HZAU (HZAUMO-2024-0028). The mice were housed in a temperature-controlled environment (22 ± 2 °C) with humidity maintained at 40–60% and subjected to a light/dark cycle of 12 h each. They had unrestricted access to food and water during the acclimatization phase. After a one-week adaptation period, all mice were weighed and divided into four groups. Three groups received daily oral administration of PFO at doses of 2.0, 5.0, and 10.0 mg/kg.bw for one week, while the control group received an equivalent volume of saline solution. Subsequently, the animals were euthanized under anesthesia. A portion of the intestinal tissue was rapidly frozen in liquid nitrogen and stored at −80 °C for further analysis, while the remaining tissue was fixed in a solution containing 4% paraformaldehyde for histopathological examination.

### 2.5. Transepithelial Electrical Resistance Determination

The IPEC-J2 cells (4 × 10^4^ cells/insert) were cultured in the upper compartment of 24 collagen-coated Millicell filter inserts with a pore size of 3.0 mm (Corning Inc., Corning, NY, USA). The lower compartment was filled with DMEM supplemented with 10% FBS. Trans-epithelial electrical resistance (TEER) was determined by measuring the potential difference between the apical and basolateral sides using an epithelial tissue volt-ohm-meter. Once a stable resistance level was reached, PFO at various concentrations (0.1 mg/L, 0.2 mg/L, 0.5 mg/L, and 1.0 mg/L) was added to the apical compartments of the transwell chambers. The presented data represents the average of triplicate samples from three independent real-time experiments. Resistance was calculated in both Ω/cm^2^ and as a percentage of the original TEER value compared to the control using this formula: normal resistance (Ω/cm^2^) = (TEER − blank)/0.33 cm^2^ (for 24-well Millicell filters).

### 2.6. The Impact of PFO on Cell Permeability Assessed by FD-4 Infiltration

To assess the permeability of IPEC-J2 cell monolayers following PFO treatment, a final concentration of 1.0 mg/mL of FD-4 (FITC-Dextran 4) was introduced into the apical compartment of transwell plates after exposure to various concentrations of PFO. Subsequently, the medium from the basolateral compartment was collected and gently mixed after a 2-h incubation period, followed by the transfer to 96-well black opaque plates (100 μL per well, in triplicate). The fluorescent intensity (excitation at 485 nm; emission at 528 nm) was measured using a Spark multimode microplate reader (Tecan Trading AG, Männedorf, Switzerland). The recorded fluorescent intensity served as an indicator for assessing the permeability of the IPEC-J2 monolayer.

### 2.7. CCK-8 Assay

The CCK-8 assay was performed using a CCK-8 kit from Beyotime (Shanghai, China), following the manufacturer’s instructions. Initially, IPEC-J2 cells were seeded in 96-well plates at a density of 5 × 10^3^ cells per well in 100 μL of culture medium and incubated for 12 h. Subsequently, the cells were treated with various concentrations of PFO and then exposed to 10 μL of CCK-8 solution at 37 °C for 30 min. In each experiment, a culture solution control (consisting of DMEM medium and CCK-8) and a blank control (comprising cells, DMEM medium, and CCK-8) were concurrently established. The plate was then placed in a microplate reader (PE Enspire, Waltham, MA, USA) to measure the optical density (OD) at 450 nm/630 nm wavelength. Each experiment was repeated three times within each group.

### 2.8. Mitochondrial Membrane Potential Monitoring

The mitochondrial membrane potential (MMP) was assessed utilizing a JC-1 Mitochondrial Membrane Potential Detection Kit (Beyotime, C2003S). For the assessment, 1 mL of JC-1 staining working solution was added to each well of a 6-well cell plate and mixed thoroughly. The plate was then incubated at 37 °C for 20 min in a cell incubator. Subsequently, the supernatant was removed, and the cells were washed twice with JC-1 staining buffer. Following this, the cells were treated with PFO for 30 min in the dark at 37 °C. The images of mitochondrial membrane potential were captured using a confocal microscope (Zeiss LSM 880, Zeiss, Oberkochen, Germany). The fluorescence intensity was measured using a multifunctional microplate reader (PE Enspire, Waltham, MA, USA).

### 2.9. LDH Assay

The LDH release was quantified using the LDH Release Assay Kit (Beyotime, C0017), and the absorbance was measured at 450 nm with a universal microplate reader (PE Enspire, Waltham, MA, USA). The percentage of LDH release was calculated using the following formula: LDH release (%) = (absorbance of the treated sample − absorbance of the sample control well)/(absorbance of the cell’s maximum enzyme activity − absorbance of sample control well) × 100.

### 2.10. Cell Membrane Staining

To investigate the effects of PFO on cell membranes, DiO-dye staining experiments were conducted. DiO, a widely utilized fluorescent probe, is a lipophilic membrane dye that diffuses laterally upon entering the cell membrane, gradually staining the entire membrane [29]. Subsequently, the DiO Cell Membrane Staining Kit (Beyotime, C1038) was employed to assess the integrity of the cell membrane following the manufacturer’s guidelines. The cells were then imaged under a fluorescence microscope at 37 °C after 30 min of PFO treatment.

### 2.11. Reactive Oxygen Species (ROS) Detection

The intracellular levels of ROS were evaluated using the ROS Assay Kit (Beyotime, S0033). IPEC-J2 cells were seeded in a 12-well culture plate at a density of 1 × 10^5^ cells per well. After PFO treatment, the cells were washed twice with PBS and then exposed to 10 μM DCFH-DA at 37 °C for 30 min under light-free conditions. Subsequently, fluorescence signals were captured using fluorescence microscopy with excitation/emission wavelengths set at 488 nm/521 nm. The fluorescence intensity was measured using a multifunctional microplate reader (PE Enspire, Waltham, MA, USA).

### 2.12. RNA Extraction and RT-qPCR

The conventional TRIzol method (Thermo Fisher Scientific, Waltham, MA, USA) was employed to extract total RNA from tissues or cells. Subsequently, the concentration of RNA was determined using a Nanodrop 8000 nucleic acid protein analyzer, and the results were recorded. Total RNA was reverse transcribed following the protocols of HiScript II Q RT SuperMix for qPCR gDNA wiper (Vazyme, Nanjing, China). Quantitative PCR using SYBR Green was conducted on the QuantStudio™ Detection System (Thermo, Waltham, MA, USA) in accordance with the manufacturer’s instructions. Normalization of target gene expression was conducted by employing β-actin as a reference gene and calculated using the 2^−ΔΔCT^ method. The primer sequences used in the qPCR assays can be found in Table 1.

### 2.13. Western Blot

The IPEC-J2 cells or tissue samples were subjected to protein extraction using pre-chilled RIPA buffer (Beyotime, Shanghai, China). The protein concentration was determined using the BCA protein assay kit (Beyotime, Shanghai, China). Subsequently, the tissue homogenates or cell lysates were diluted in a loading buffer and boiled at 100 °C for 10 min. The proteins were then separated by SDS-PAGE and transferred onto polyvinylidene difluoride (PVDF) membranes. Following this step, the PVDF membranes were blocked with a solution of 5% bovine serum albumin (BSA) dissolved in 10 mM Tris-buffered saline with 0.05% Tween 20 (TBST) for a duration of 2 h. Next, the membranes were incubated overnight at 4 °C with primary antibodies. After washing and subsequent incubation with horseradish peroxidase-conjugated secondary antibodies, the blots underwent another round of washing before visualizing the signals utilizing an ECL kit from Bio-Rad Laboratories (Hercules, California, USA). 

### 2.14. Immunofluorescent Staining

After treatment with PFO, the cells were initially washed with PBS and subsequently fixed in 4% paraformaldehyde for 30 min at room temperature. They were then permeabilized with 1% Triton X-100 for 20 min at room temperature. Following this step, the cells underwent blocking with 5% bovine serum albumin for 1 h at room temperature before being incubated overnight at 4 °C with primary antibodies. After thorough washing, the cells were subjected to incubation with Cy3-labeled anti-rabbit IgG secondary antibody for 1 h at room temperature and finally mounted using DAPI (Beyotime, Shanghai, China) for fluorescence imaging. The imaging process was conducted using a confocal microscope (Zeiss LSM 880, Zeiss, Oberkochen, Germany).

### 2.15. Statistical Analysis

The data were obtained from a minimum of three independent experiments. Statistical analyses were performed using a one-way ANOVA in GraphPad Prism 9.0 software, and pairwise comparisons were further assessed using a *t*-test. The mRNA expression and protein levels were presented as fold changes relative to the control group. Statistical significance was determined through a one-way ANOVA, followed by a Tukey post-hoc test. Significance levels were indicated as * for *p* < 0.05, ** for *p* < 0.01, and *** for *p* < 0.001.

## 3. Results

### 3.1. Expression and Purification of Theta Toxin (PFO)

**Figure 1:**
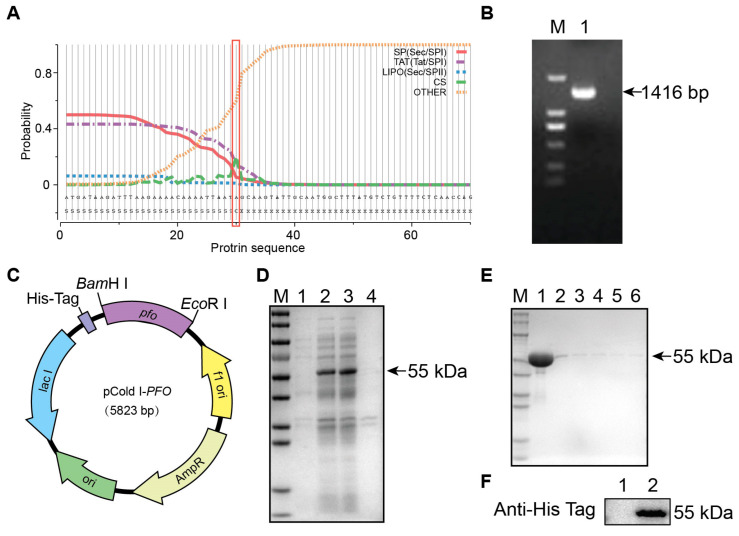
Expression and purification of theta toxin (PFO) (**A**): Signal peptide of PFO proteins using SignalP-5.0 analysis. SP (Sec/SPI): type of signal peptide predicted; CS: the cleavage site; Other: the probability that the sequence does not have any kind of signal peptide. (**B**): Amplification of *PFO* genes shows the predicted fragment size after separation on a 1.0% agarose gel. M: DNA DL2000 Marker; lane 1: *PFO* gene. (**C**): Construction map of the plasmid pCold-1-*PFO*. (**D**): SDS-PAGE electrophoresis diagram of PFO protein-induced expression. Lane M: standard protein molecular weight marker; line 1: uninduced protein; lane 2: induced protein whole bacteria after ultrasound; lane 3: induced protein supernatant after ultrasound; lane 4: induced protein precipitation after ultrasound. (**E**): Purification of PFO under different conditions. Lane M: standard protein molecular weight marker; lane 1: 100 mM of imidazole elution fraction, lane 2: 150 mM of imidazole elution fraction, lane 3: 200 mM of imidazole elution fraction, lane 4: 250 mM of imidazole elution fraction, lane 5: 300 mM of imidazole elution fraction, lane 6: 500 mM of imidazole elution fraction. (**F**): Western blot analysis of purified PFO protein. Lane 1: negative control; lane 2: purified PFO protein.

Initially, the SignalP 5.0 server was used to predict the signal peptide and its cleavage site in the PFO protein. The analysis identified a signal-peptide cleavage site at position 30 (Figure 1A). Subsequently, specific primers were designed to amplify PFO fragments. Gel electrophoresis confirmed the size consistency between the amplified and predicted fragments (Figure 1B). Sequencing of the PCR products revealed a sequence identical to those in GenBank under accession numbers CP028149.1, CP102300.1, and DQ673099.1. The study utilized pCold-I vectors, which contain the *cspA* gene responsible for protein expression at low temperatures to prevent insolubility, for cloning the *PFO* gene. The fragment was ligated into the pCold-I vector using *Bam*H I and *Eco*R I restriction sites, resulting in the creation of a new expression vector called pCold-I-*PFO* (Figure 1C). Transformation of *E. coli* BL21 (DE3) cells with the pCold-I-*PFO* vector led to the expression of the PFO protein in the supernatant after cell lysis (Figure 1D). Subsequent purification and SDS-PAGE analysis revealed a single band at approximately 55 kDa (Figure 1E). Western blot analysis further confirmed the successful purification of the PFO protein (Figure 1F). The protein concentration was quantified as 12.0 mg/mL using the BCA method.

### 3.2. PFO Exposure Induces Intestinal Epithelial Barrier Damage In Vivo

**Figure 2:**
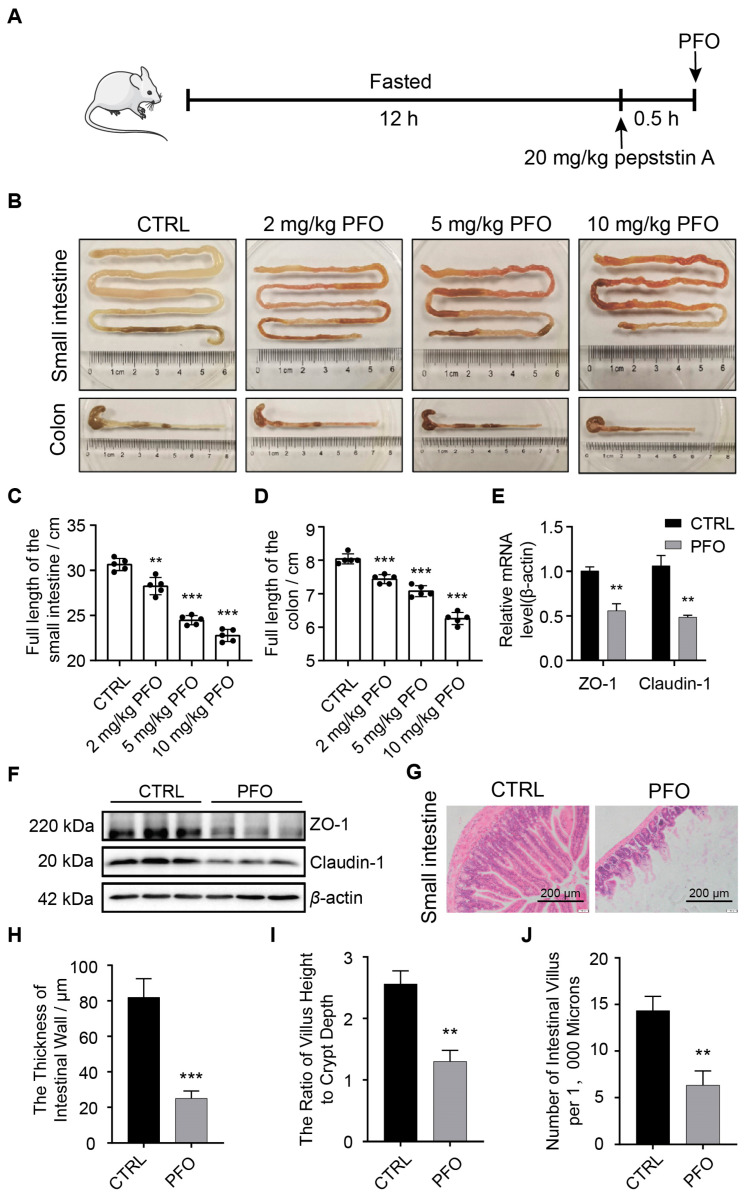
PFO exposure induces intestinal epithelial barrier damage in vivo. (**A**): Experimental design and scheme of the animal treatments. (**B**–**D**): Representative small intestine and colon photos were captured using a camera, and the length of the small intestine and colon were measured (n = 6). (**E**): Relative mRNA expressions of ZO-1 and claudin-1 in the control and PFO groups (n = 3). (**F**): Relative protein abundances of ZO-1, claudin-1, and β-actin in the small intestine of mice after PFO exposure (n = 3). (**G**): Representative images of H&E-stained small intestine sections in the control and PFO groups (scale bar 200 μm). (**H**–**J**): Statistical analysis of the thickness of the intestinal wall, the ratio of villus height to crypt depth, and the number of intestinal villi per 1000 microns in the control and PFO groups. An ANOVA followed by a two-tailed unpaired Student’s *t*-test was used to compare the different groups. All data were presented as mean ± SEM, ** *p* < 0.01, *** *p* < 0.001.

In the study, mice were orally administered PFO at dosages of 2, 5, and 10 mg/kg/day via gavage for seven consecutive days. The control group received normal saline at the same volume by gavage once daily for seven consecutive days. The results depicted in Figure 2B–D show that PFO treatment caused a significant decrease in the lengths of the small intestine and colon in a dose-dependent manner when compared to the control group that did not receive treatment. These observations suggest that PFO induced dose-dependent changes in general symptoms of balb/c mice, including alterations in stool consistency, as well as shortening of the small intestine and colon. Claudin-1 and zonula occludens-1 (ZO-1) are key proteins involved in maintaining mechanical barrier function. Intestinal tissue analysis using RT-qPCR and Western blotting showed reduced mRNA and protein levels of ZO-1 and Claudin-1 in the small intestine after PFO treatment (Figure 2E,F). Histological examination with HE staining was performed to evaluate the effects of PFO on intestinal morphology and pathology in treated mice. In the control group, the intestinal villi exhibited a well-organized structure, with neatly arranged crypts, orderly cell alignment, clear morphology, and no apparent damage (Figure 2G). In contrast, PFO treatment led to a significant decrease in intestinal villus height, the ratio of villus height to crypt depth, and intestinal wall thickness compared to the control group (Figure 2H,I). These results collectively suggest that PFO can disrupt the function and integrity of intestinal structures in vivo in a dose-dependent manner.

### 3.3. PFO Exposure Induces Damage to the Intestinal Epithelial Barrier Model In Vitro

**Figure 3:**
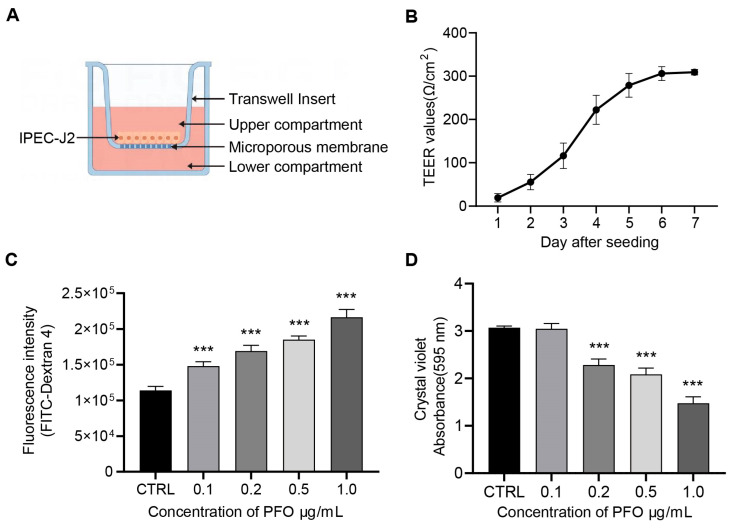
PFO exposure induces damage to the intestinal epithelial barrier model in vitro (**A**): Schematic diagram of a conventional transwell plate with the IPEC-J2 cell monolayer. (**B**): TEER values of the IPEC-J2 cell monolayers. (**C**): Fluorescence intensity in the lower compartment of IPEC-J2 cell monolayers treated with different concentrations of PFO. (**D**): Measured by absorbance at 595 nm of crystal violet-stained biofilms after treatment with different concentrations of PFO. *** *p* < 0.001.

The study aimed to investigate the impact of PFO on intestinal barrier function using in vitro models of well-differentiated intestinal porcine epithelial cell (IPEC-J2) monolayers in transwell plates (Figure 3). Intestinal barrier function was assessed by measuring the trans-epithelial electrical resistance (TEER) in IPEC-J2 cell monolayers cultured on polyester membranes in transwell inserts (Figure 3A). After 7 days of culture, the TEER value of the epithelial cells reached a plateau, with the highest value being approximately 300 Ω/cm^2^, indicating the formation of a dense intestinal epithelial barrier by the cultured cells (Figure 3B). Different concentrations of PFO were applied to the upper chamber and incubated at 37 °C for 30 min. Following incubation, the PFO solution was removed, and both the upper and lower chambers were washed four times with 200 μL of pre-heated D-Hank solution. FITC-dextran (4 kDa, 1 mg/mL) was then introduced into the upper chamber, and the fluorescence intensity in the lower chamber was measured after 2 h (n = 5) (Figure 3C). Additionally, crystal violet was added to the transwell chamber to stain cells at room temperature for 30 min. The transwell inserts were rinsed with PBS to eliminate unbound crystal violet and air-dried. The crystal violet bound to the cells was dissolved in 10% acetic acid, and the absorbance at 595 nm was recorded (Figure 3D). The experimental results showed that increasing PFO concentrations led to higher fluorescence intensity in the lower chamber, while the absorbance of crystal violet at 595 nm decreased with higher PFO concentrations. These findings suggest that PFO could dose-dependently compromise the intestinal epithelial barrier in vitro. 

### 3.4. PFO Exposure Influences IPEC-J2 Cell Activity and Intercellular Tight Junction Impairment

**Figure 4:**
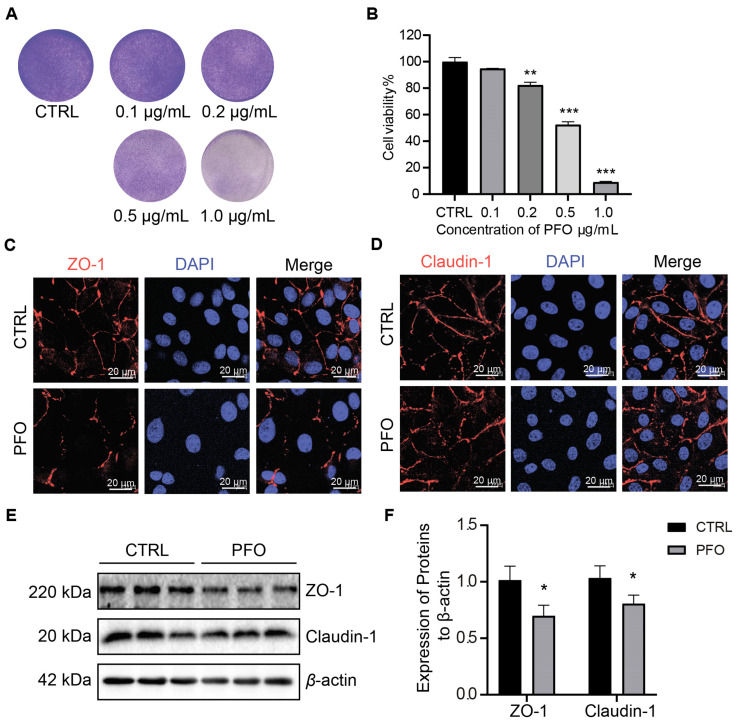
PFO exposure influences IPEC-J2 cell activity and intercellular tight junction impairment (**A**): IPEC-J2 cells were treated with different concentrations of PFO (0.5 μg/mL for 30 min) and with crystal violet staining. (**B**): IPEC-J2 cells were treated with different concentrations of PFO, and the viability of the cells was determined by the CCK-8 assay. (**C**,**D**): Expressions of ZO-1 and Claudin-1 were determined by immunofluorescence assay under the laser scanning confocal microscope. ZO-1 (red) and Claudin-1 (red) localized at the IPEC-J2 cells intercellular. The continuous distribution of ZO-1 and claudin-1 in IPEC-J2 of the control group (scale bar 20 μm). (**E**): Relative protein abundances of ZO-1, claudin-1, and β-actin in IPEC-J2 after PFO exposure (0.5 μg/mL for 30 min) (n = 3). (**F**): Quantification of protein relative to control in (**E**) normalized to β-actin protein level. * *p* < 0.05, ** *p* < 0.01, *** *p* < 0.001.

To assess cell viability, IPEC-J2 cells were exposed to different concentrations of PFO (0 μg/mL, 0.1 μg/mL, 0.2 μg/mL, 0.5 μg/mL, and 1.0 μg/mL) for 30 min. Subsequently, crystal violet staining and CCK-8 analysis were performed. The crystal violet assay indicated a significant cytotoxic effect of PFO on the cells, which was further confirmed by the CCK-8 assay (Figure 4A,B). These results highlight the dose-dependent impact of PFO, with minimal effects observed in the control group. Immunofluorescence staining for claudin-1 and ZO-1 was used to evaluate intestinal barrier integrity. Compared to the control group, the PFO-treated group showed evident disruption of cell membranes (Figure 4C,D). Meanwhile, PFO also reduced the protein expression of ZO-1 and Claudin-1 in IPEC-J2 monolayer cells (Figure 4E,F). In conclusion, these findings suggest that PFO can cause damage to intestinal epithelial cells, leading to compromised mechanical integrity of the intestinal epithelial barrier.

### 3.5. PFO Causes Mitochondrial Damage and Increased ROS Content

**Figure 5:**
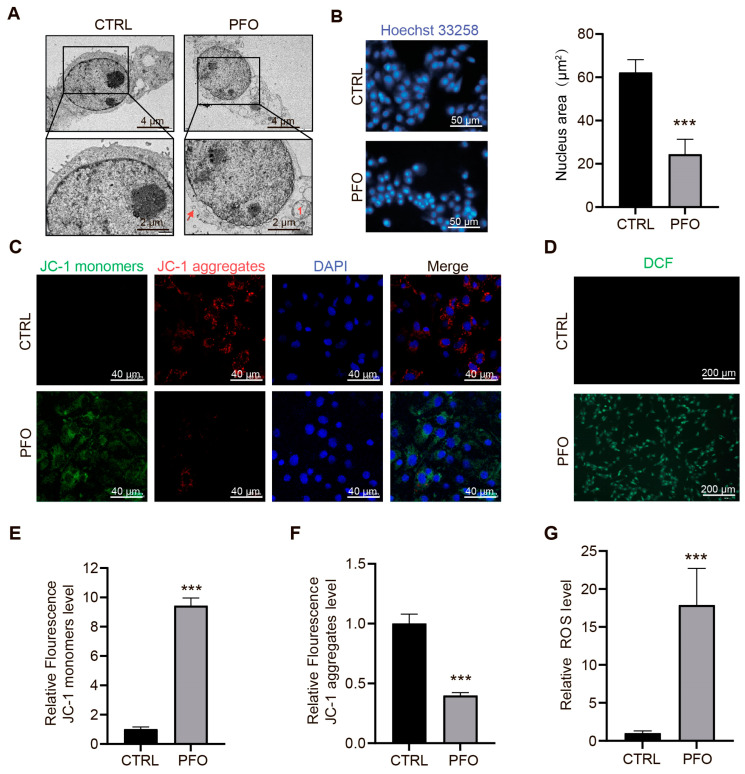
PFO causes mitochondrial damage and increased ROS content (**A**): Transmission electron microscopy. The red arrowheads indicate the emerging pore from the plasma membrane (scale bar 4 μm), The red number 1 indicates mitochondria. (**B**): Hoechst staining of cells treated with PFO (0.5 μg/mL for 30 min) (scale bar 50 μm). (**C**): Fluorescence image of IPEC-J2 cells stained with JC-1. Red fluorescence indicates JC-1 aggregation in healthy mitochondria, whereas green fluorescence indicates cytosolic JC-1 monomers indicative of mitochondrial membrane potential (MMP) collapse. Merged images indicated co-localization of JC-1 aggregates and monomers (scale bar 40 μm). (**E**,**F**): A multifunctional microplate reader was used to detect the relative levels of JC-1 monomeric green fluorescence and JC-1 polymeric red fluorescence intensities. (**D**): Evaluation of ROS production in IPEC-J2 treated with PFO (0.5 μg/mL for 30 min) using DCF (scale bar 200 μm). (**G**): In situ fluorescence detection of intracellular ROS. ROS level was detected using the DCF probe of a multifunctional microplate reader. *** *p* < 0.001.

To evaluate subcellular indicators of cell damage, transmission electron microscopy (TEM) was utilized to assess the integrity of the nuclear envelope and mitochondrial morphology. The TEM analysis revealed that PFO-induced mitochondrial damage was characterized by mitochondrial swelling. Additionally, observations of cell membrane pores were made alongside nuclear shrinkage (Figure 5A). Following the TEM examination, measurements were taken on the nuclear morphology and mitochondrial membrane potential of IPEC-J2 cells post-PFO treatment. As shown in Figure 5B, hoechst33258 staining indicated smaller nuclei in the PFO group compared to the control group. The assessment of mitochondrial membrane potential was conducted using the JC-1 Mitochondrial Membrane Potential Assay Kit. The PFO-treated cells exhibited a significant increase in green fluorescent intensity and a decrease in red fluorescence. The red JC-1 aggregates represent high mitochondrial membrane potential, while the green JC-1 monomers indicate low mitochondrial membrane potential (Figure 5C,E,F). Detection of PFO-induced reactive oxygen species (ROS) production was carried out using the DCF-DA reagent, a fluorescent probe for visualizing ROS. The results showed a marked increase in green fluorescence due to PFO treatment, contrasting with the minimal green fluorescence observed in the absence of PFO treatment (Figure 5D,G). These findings suggest that PFO not only reduces mitochondrial membrane potential but also increases ROS production, thereby concurrently disrupting the IPEC-J2 cell membrane.

### 3.6. PFO Induces Pyroptosis in IPEC-J2 Cells

**Figure 6:**
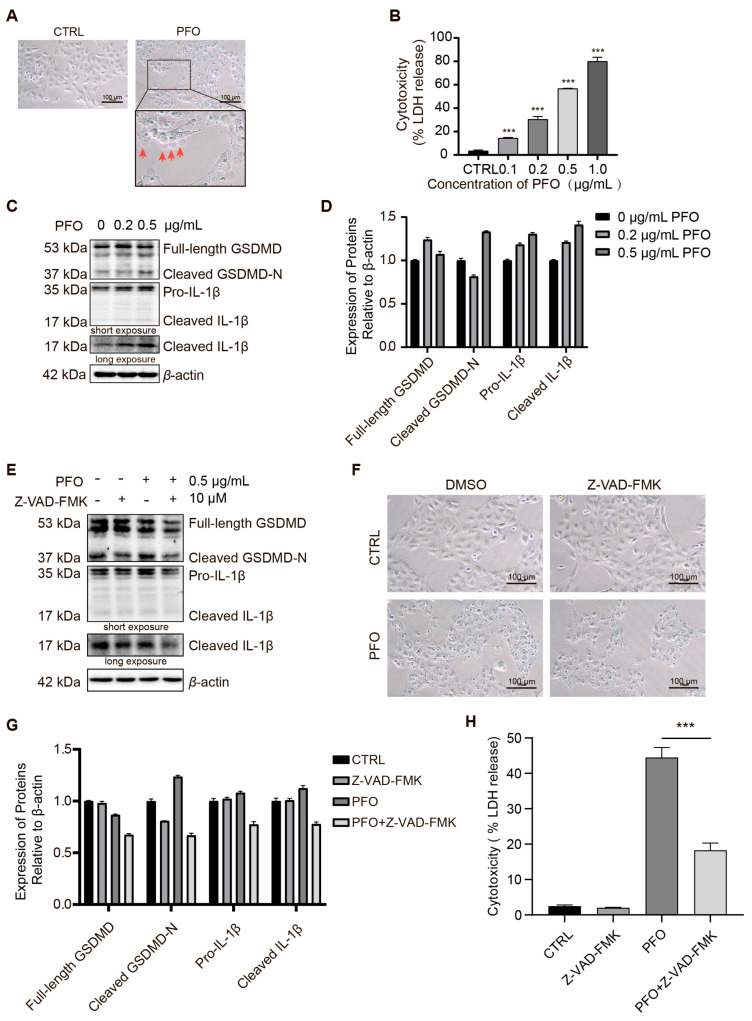
PFO induces pyroptosis in IPEC-J2 cells (**A**): Microscopic imaging of IPEC-J2 cells after PFO (0.5 μg/mL for 30 min) treatment (scale bar 100 μm), the red arrows indicate bubbles on the surface of IPEC-J2 cells. (**B**): Lactic dehydrogenase (LDH) release was assessed after exposure to different concentrations of PFO for 30 min. (**C**): Expression of GSDMD and IL-1β in IPEC-J2 cells after PFO treatment. (**D**): Quantification of protein relative to control in (**C**) normalized to β-actin protein level. (**E**): Treatment of Z-VAD-FMK at a dose of 10 µM inhibits the activation of GSDMD and IL-1β by PFO (0.5 µg/mL for 30 min). (**F**): Z-VAD-FMK partially prevented morphological alterations induced by PFO (0.5 μg/mL for 30 min) (scale bar 100 μm). (**G**): Quantification of protein relative to control in (**E**) normalized to β-actin protein level. (**H**): Lactic dehydrogenase (LDH) release was assessed after exposure to different treatments for 30 min. *** *p* < 0.001.

To evaluate the effects of purified PFO on cultured epithelial cell monolayers, we treated IPEC-J2 cells with growing concentrations of PFO. IPEC-J2 cells began bubbling in just 15 min (Figure 6A). With increasing PFO concentrations in the cell cultures, the LDH release of IPEC-J2 cells increased significantly (Figure 6B). Additionally, western blot analysis analyses showed that PFO treatment upregulated the expression of cleaved-GSDMD and cleaved-IL-1β in a dose-dependent manner (Figure 6C,D). The above results indicate that PFO triggers GSDMD-mediated IPEC-J2 cell pyroptosis. Pyroptosis is a lytic type of programmed cell death that was traditionally associated with the involvement of inflammatory caspases [30]. Z-VAD-FMK (Z-VAD), a pan-caspase inhibitor, was used to inhibit caspase activation. To study whether PFO-induced pyroptosis is mediated by the activation of caspases, we used Z-VAD-FMK and demonstrated that blocking caspase activity can partially inhibit PFO-induced pyroptosis (Figure 6E,G). However, PFO treatment cannot significantly inhibit pyroptosis of IPEC-J2 cells in vitro (Figure 6F,H). This result shows that PFO could induce pyroptosis by other pathways.

**Figure 7:**
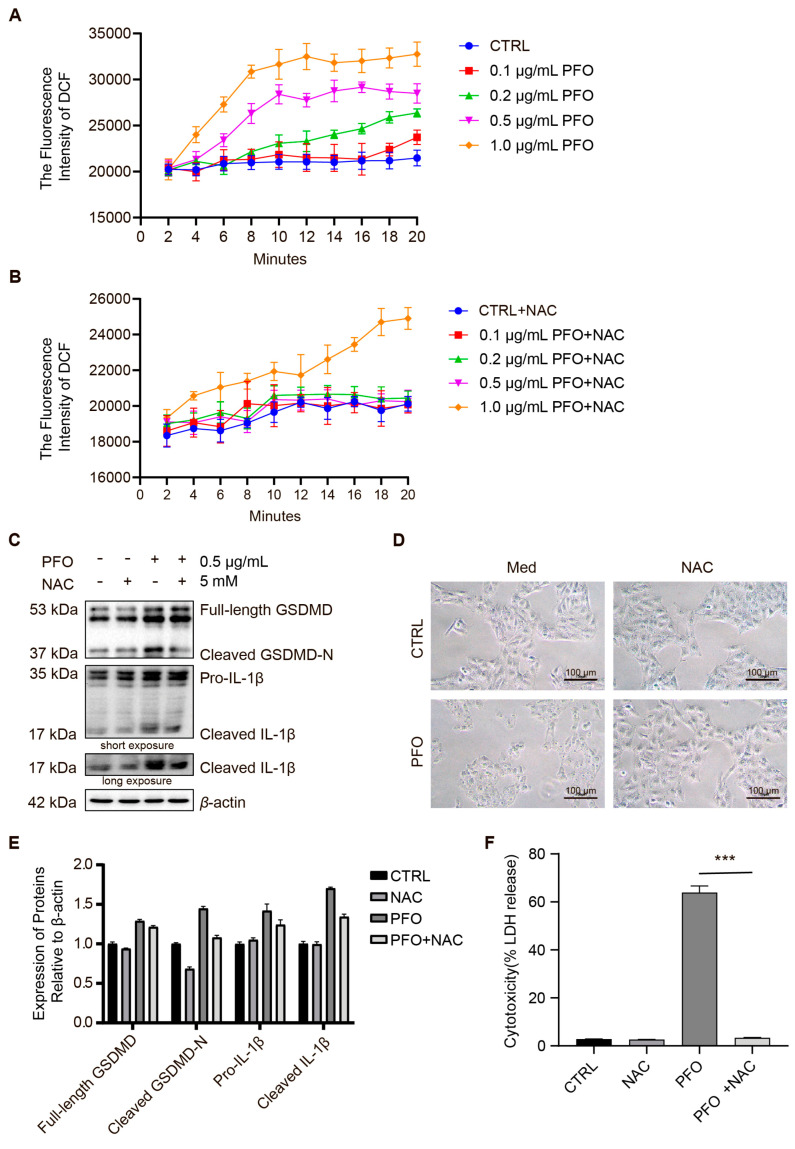
NAC inhibits PFO-induced pyroptosis in IPEC-J2 cells (**A**): Different concentrations of PFO-induced ROS production in IPEC-J2 cells. (**B**): NAC inhibits PFO-induced ROS production in IPEC-J2 cells. (**C**): Treatment of NAC at a dose of 5 mM inhibits the activation of GSDMD and IL-1β by PFO (0.5 μg/mL for 30 min). (**D**): NAC prevented morphological alterations induced by PFO (0.5 μg/mL for 30 min) (scale bar 100 μm). (**E**): Quantification of protein relative to control in (**C**) normalized to β-actin protein level. (**F**): Lactic dehydrogenase (LDH) release was assessed after exposure to different treatments for 30 min. *** *p* < 0.001.

In a previous experimental study, we showed that PFO induces mitochondrial damage and ROS production. Over time, varying concentrations of PFO were found to increase ROS production in IPEC-J2 cells (Figure 7A). Furthermore, NAC was shown to inhibit ROS generation induced by low doses of PFO in IPEC-J2 cells (Figure 7B). To explore the role of ROS in the PFO-induced pyroptosis in IPEC-J2 cells, we used ROS scavenger N-acetyl-cysteine (NAC) to inhibit ROS production. NAC reduces PFO-induced cleaved-GSDMD and cleaved-IL-1β expression (Figure 7C,E). In addition, NAC inhibited PFO-induced bubble production in IPEC-2 cell membranes (Figure 7D). NAC significantly inhibited PFO-induced release of LDH (Figure 7F). These results indicate that PFO enhances pyroptosis through the ROS-GSDMD signaling in IPEC-J2 cells.

## 4. Discussion

Infection with *C. perfringens* a significant foodborne pathogen globally, can cause a range of inflammatory diseases, presenting a major challenge to the healthy development of the global livestock farming industry. *C. perfringens* has the ability to cause diseases in various species, including humans, birds, poultry, pigs, dogs, and goats [31]. With high morbidity, disability, and mortality rates, a *C. perfringens* infection primarily impacts the digestive system. The ban on in-feed antibiotics in many countries has led to an increase in cases of NE, an enterotoxin disease caused by *C. perfringens*. However, the exact mechanism remains unclear, highlighting the need for further research to provide new insights for disease management.

*C. perfringens* is known to cause various conditions such as food poisoning, gas gangrene, and antibiotic-associated diarrhea through the production of multiple toxins and extracellular enzymes. These toxins and enzymes can affect not only the nervous system [32], digestive system [33], and blood system [34] but also other bodily systems [35]. One of the key cytolytic toxins produced by *C. perfringens* is PFO, a 54 kDa toxin that binds to cholesterol, creating pores in the host cell membrane. PFO, along with another common toxin, plays a crucial role in the formation of local lesions associated with gangrene/malignant edema in both humans and animals [26,36]. Despite this, there is limited literature on the specific mechanisms underlying damage to the intestinal epithelial barrier. The findings of this study suggest that exposure to PFO leads to the depolarization of mitochondrial membrane potential, resulting in the excessive generation of ROS and ultimately causing pyroptosis in IPEC-J2 cells.

Pyroptosis is a recently recognized form of programmed inflammatory cell death that is activated through pathways mediated by classic caspase-1 inflammasomes or non-classical caspase-4, caspase-5, and caspase-11 [37]. However, the PFO-induced pyroptosis observed in IPEC-J2 cells in this study was both caspase-dependent and independent of caspases. Notably, the treatment with the pan-caspase inhibitor Z-VAD-FMK did not completely inhibit PFO-induced pyroptosis, suggesting the involvement of other factors influencing pyroptosis in IPEC-J2 cells concurrently. The formation of cell membrane bubbles is likely a result of the pressure disparity between the interior and exterior of the cell membrane caused by the pore formation from PFO [38]. The study further revealed that PFO led to mitochondrial dysfunction characterized by a reduction in mitochondrial membrane potential and an increase in ROS production. ROS have garnered significant attention from researchers due to their involvement in cell death when produced in excess [39,40,41]. The cellular redox state thus plays a critical role in GSDMD activities, with ROS acting as enhancers of GSDMD pore-forming activities [42]. Subsequently, an investigation was conducted to determine whether NAC treatment could protect IPEC-J2 cells by reducing intracellular ROS levels. The inhibition of ROS production with NAC suppressed PFO-induced GSDMD activation and pyroptosis in IPEC-J2 cells. Hence, mitigating oxidative stress is imperative for preventing PFO-induced pyroptosis in IPEC-J2 cells.

The intestinal epithelial barrier is considered the foremost barrier and plays a crucial role in preventing pathogen penetration. Pathogens are unable to breach the epithelial barrier under normal circumstances. However, during microbial infection, pathogens can interact with and harm the epithelial barrier, resulting in increased intestinal permeability. This phenomenon can trigger enteritis and diarrhea [43,44]. ZO-1 and ZO-2 are expressed in various tissue cells, but their expression patterns may vary. ZO-1 is found in a wide range of cell types, while ZO-2 is present in specific cells. Both Claudin-1 and Claudin-5 belong to the tight junction protein family, which is crucial for maintaining the structure and function of intercellular tight junctions. Claudin-1 is broadly expressed in different epithelial cells such as intestinal, renal, and respiratory epithelial cells. On the other hand, Claudin-5 is predominantly found in vascular endothelial cells, particularly in brain microvascular endothelial cells. As a result, we selected ZO-1 and Claudin-1 as markers for testing intestinal barrier function. Pyroptosis of intestinal epithelial cells induced by pathogens disrupts the functions of the intestinal barrier. The induction of pyroptosis in IPEC-J2 cells by PFO results in the dysfunction of the intestinal epithelial barrier. Targeting endothelial cells with CPB causes damage to these cells by compromising the integrity of the intestinal epithelial barrier [45,46]. Additionally, the *C. perfringens* ETX toxin can breach the circulatory system through the compromised intestinal epithelial barrier, leading to dysfunction of the blood–brain barrier and subsequent neurotoxic effects [47]. PFO has been demonstrated to act synergistically with the α-toxin in gas gangrene and hemorrhagic enteritis [48]. 

However, PFO exhibits a dual nature. Recently, researchers have developed a couple of valuable biosensors that allow for the visualization of cholesterol in cellular membranes using domain 4 (D4) of PFO, which is a cholesterol-binding toxin [49]. Hemolysin BL shares the pore-forming capability of PFO. Additionally, future studies should outline specific therapeutic strategies for targeting tumors with PFO [50].

## 5. Conclusions

Our study highlights that *C. perfringens* PFO diminishes mitochondrial membrane potential, resulting in heightened ROS release and the creation of an oxidative stress environment. This oxidative stress setting promotes GSDMD-mediated IPEC-J2 pyroptosis. Pyroptosis results in the shedding of intestinal epithelial cells, compromising the integrity of the intestinal epithelial barrier. Additionally, the cell membrane alterations induced by pyroptosis disrupt the distribution of intercellular tight junction proteins, further compromising the intestinal barrier integrity. This compromised barrier allows gut microbes to breach the barrier, triggering severe inflammation and ulceration. Additionally, NAC shows promise in alleviating PFO-induced damage in IPEC-J2 cells.

## Figures and Tables

**Table 1:** Sequences of primers used for qRT-PCR.

Gene	Forward Primer Sequence (5′ to 3′)	Reverse Primer Sequence (5′ to 3′)
*ZO-1*	ACCCGAAACTGATGCTGTGGATAG	AAATGGCCGGGCAGAACTTGTGTA
*Claudin-1*	AGCTGCCTGTTCCATGTACT	CTCCCATTTGTCTGCTGCTC
*β-actin*	AAATCGTGCGTGACATCAAA	ATGCCACAGGATTCCATACC

## Data Availability

The data presented in this study are available upon request from the corresponding author.
